# Emergency Awake Abdominal Surgery Under Thoracic Epidural Anaesthesia in a High-Risk Patient Within a Resource-Limited Setting

**DOI:** 10.7759/cureus.34856

**Published:** 2023-02-11

**Authors:** Johannes J Le Roux, Koji Wakabayashi, Zainub Jooma

**Affiliations:** 1 Anaesthesiology, Chris Hani Baragwanath Academic Hospital, Johannesburg, ZAF; 2 Anaesthesia and Critical Care, Charlotte Maxeke Johannesburg Academic Hospital, Johannesburg, ZAF; 3 Anaesthesiology, University of the Witwatersrand, Johannesburg, Johannesburg, ZAF

**Keywords:** emergency abdominal surgery, loco-regional anaesthesia, surgical stress response, thoracic epidural analgesia, awake laparotomy

## Abstract

Awake abdominal surgery is performed daily around the world for caesarean section surgery under lumbar subarachnoid anaesthesia and/or graded lumbar epidural anaesthesia. Reports of awake abdominal surgery under thoracic epidural anaesthesia (TEA) for patients with bowel obstruction are scarce, as this patient population is at high risk for pulmonary aspiration. In this report, we describe a case in which a graded TEA was successfully used as the sole anaesthetic technique in a patient with severe pulmonary disease undergoing an awake emergency laparotomy for bowel ischaemia for whom no postoperative intensive care monitoring was available. No anaesthetic or surgical complications occurred, and the patient was discharged home seven days after the surgical procedure. A 30-day follow-up revealed no residual anaesthetic or surgical complications, with a return to baseline function.

## Introduction

Emergency laparotomy is a common surgical procedure that carries a high risk of morbidity and mortality. In high-risk patients, mortality for emergency abdominal surgery ranges from 14.9% to 25% [1]. It is frequently performed for patients with pre-existing diseases over and above their acute insult presenting a challenge for treating clinicians. Reports in the United Kingdom have found that patients presenting for emergency laparotomy constitute a “forgotten group” comprising a large proportion of surgical admissions, with death rates higher than in other high-income countries [1]. Acknowledgement of these risks has led to collaborations and protocols such as the National Emergency Laparotomy Audit (NELA), Emergency Laparotomy Network, and the Emergency Laparotomy Collaborative in an endeavour to identify high-risk patients and tailor perioperative care to achieve better outcomes [1]. A key component of improving outcomes has been the recognition of perioperative risk using formal risk assessments by various validated tools thereby allowing appropriate triage and expeditious care. The NELA risk assessment is one such tool validated for emergency laparotomy patients [1]. The crucial role of postoperative intensive care after emergency laparotomy has also received important appreciation and forms part of the nine key standards advocated by NELA [1].

Anaesthesia for emergency abdominal surgery is focused on minimizing the risk of pulmonary aspiration, maintaining haemodynamic stability, providing adequate analgesia, and reducing postoperative complications [1,2]. General anaesthesia (GA) with tracheal intubation and multimodal analgesia is considered the norm to achieve these goals. However, this technique increases the risk of postoperative pulmonary complications in patients with pre-existing respiratory disease.

We describe a case in which a graded thoracic epidural anaesthesia (TEA) was successfully used as the sole anaesthetic technique in a patient with severe pulmonary disease undergoing emergency laparotomy for bowel ischaemia for whom no postoperative intensive care unit (ICU) monitoring was available.

## Case presentation

A 57-year-old male patient presented with an acute bowel obstruction to the emergency department and was diagnosed with small bowel ischaemia on an abdominal computed tomography scan. He was known to have chronic obstructive pulmonary disease (COPD) secondary to a 50-pack-year smoking history and had been dependent on home oxygen for four years. On examination, he was hypoxic on room air, and an arterial blood gas demonstrated type II respiratory failure: pH of 7.33, partial pressure of oxygen (PaO2) of 51 mmHg, partial pressure of carbon dioxide (PaCO2) of 66 mmHg, standard bicarbonate (HCO3) of 36 mmol/L, lactate of 4.0 mmol/L, and a potassium of 5.8 mmol/L. He was haemodynamically stable with a mean arterial pressure (MAP) of 70 mmHg and a pulse rate of 78 beats/minute. He was not dyspnoeic when lying flat, and had a respiratory rate of 14 breaths per minute, but had bilateral wheezes on auscultation. His chest X-ray showed hyperinflated lung fields but no signs of consolidation. He was scheduled for an emergency laparotomy, but postoperative intensive care unit (ICU) care was unavailable due to limited resources.

An awake laparotomy under TEA was performed by inserting an epidural catheter at vertebral level T9/T10. In the left lateral position, the epidural space was located by a paramedian approach using a loss-of-resistance to saline technique, and the epidural catheter was secured 5 cm into the epidural space. A test dose of 3 mL 1.5% lignocaine with 1:200,000 adrenaline was administered. A T4-L2 anaesthetic level was obtained with 15 mL 0.5% bupivacaine with 1:200,000 adrenaline, given in 5 mL increments five minutes apart. The thoracic epidural was again bolused after 60 minutes with 8 mL of 0.5% bupivacaine with 1:200,000 adrenaline. No systemic analgesia was administered and epidural opioids were avoided to prevent potential respiratory embarrassment. A midline surgical incision was made extending from the T6 to L2 dermatome. The patient did not report any pain or discomfort intraoperatively.

**Figure 1:**
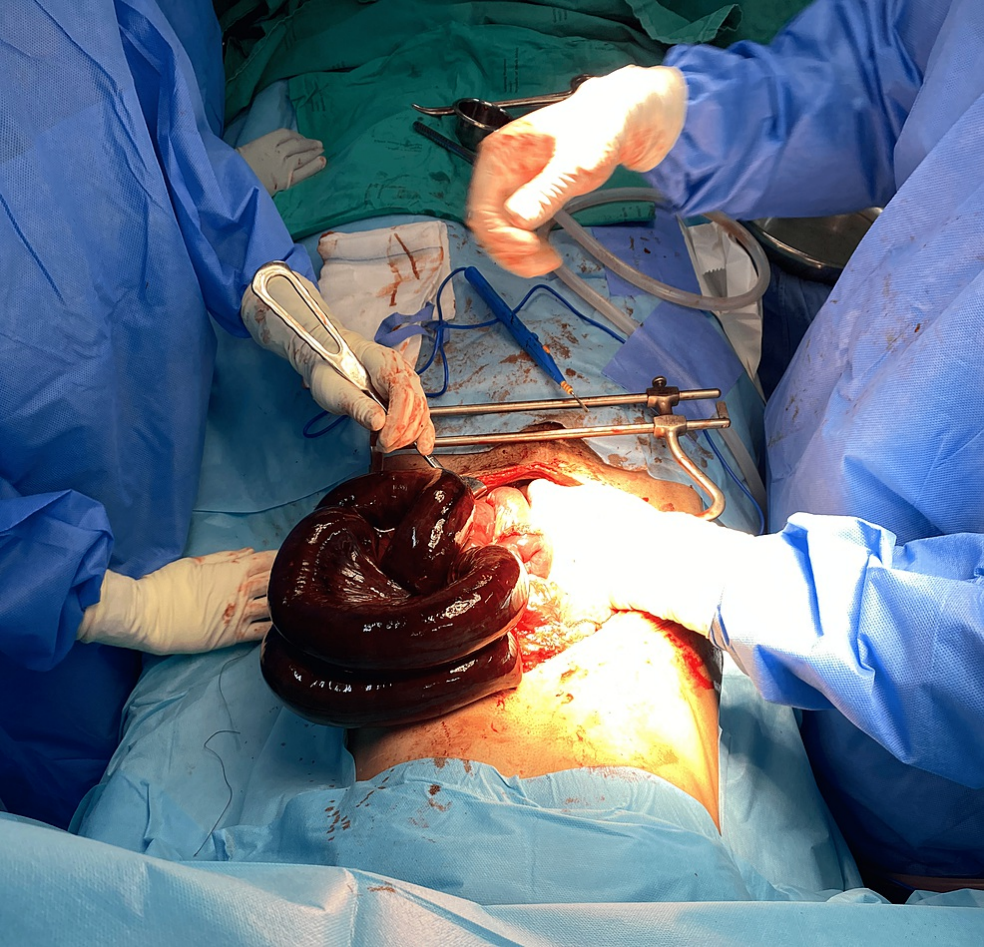
Necrotic small bowel segment

The nasogastric tube was suctioned multiple times perioperatively (total volume of 600 mL). Goal-directed fluid therapy was used to guide fluid management by monitoring static markers of cardiac output (heart rate, blood pressure, urine output, and capillary refill time). A total fluid volume of 1400 mL lactated Ringer’s solution was administered. The total blood loss was estimated to be less than 100 mL (pre and postoperative haemoglobins were 10.4 g/dL and 10.1 g/dL, respectively) and urine output averaged 1.2 mL/kg/hr. A pre-operative MAP of 70 mmHg was defended intraoperatively with a low-dose phenylephrine infusion (0.2-0.4 mcg/kg/min). For the duration of the surgery (2 hours and 13 minutes), the patient’s heart rate ranged between 64 and 81 beats/minute, MAP ranged between 70 and 81 mmHg, his serum glucose values ranged between 5.3 and 5.9 mmol/L, and his lactate levels showed a decreasing trend (2.4 mmol/L at end of surgery and 0.5 mmol/L three days post-operation). The patient's respiratory rate ranged between 12 and 16 breaths per minute during the perioperative period, and he did not show signs of respiratory embarrassment secondary to the TEA. Intraoperatively, his PaCO2 decreased to 62 mmHg and his PaO2 increased to 68 mmHg. A necrotic small bowel segment of 100 cm was resected followed by a primary anastomosis and abdominal closure (Figure 1). After surgery, the epidural catheter was removed, as the patient was transferred to a ward where TEA utilization and monitoring were not possible. He remained haemodynamically stable (MAP of 73 mmHg, heart rate of 74 beats/minute, and respiratory rate of 16 breaths/minute), and had a PaCO2 of 58 mmHg and PaO2 of 62 mmHg on room air in the immediate postoperative period. No complications were noted at follow-up, and the patient was discharged home seven days after his surgery.

## Discussion

It is well established that epidural anaesthesia and analgesia (EA) reduces postoperative complications and respiratory events and may improve long-term outcomes by attenuating the neuroendocrine stress response and by promoting earlier recovery of organ and gastrointestinal function [1]. Furthermore, EA provides superior analgesia and is associated with reduced risks of venous thromboembolism, myocardial infarction, blood loss, and renal failure [2,3]. For these reasons, EA is recognized as the gold standard for open colorectal surgery advised by the Enhanced Recovery After Surgery (ERAS) guidelines. Reviews on EA use for major abdominal surgery consistently showed clinically significant reductions in postoperative mortality, with up to 40% decreased odds of death reported [3].

In a large cohort study using the US National Surgical Quality Improvement Program (NSQIP) database where surgical procedures and type of anaesthesia were matched (264,421 patients received GA and 64,119 patients received neuraxial or regional anaesthesia), there were significantly lower odds of several postoperative complications, especially respiratory complications, and decreased hospital length of stay, but not mortality, when regional anaesthesia was compared with GA (regional analgesia supplementing GA was not accounted for). Several systematic reviews have sought to clarify the role of epidural analgesia in abdominal surgery with the consistent finding that epidural analgesia results in a reduction in rest pain after major abdominal surgery [4].

Similar ERAS care pathways for patients undergoing emergency laparotomy do not exist because of the paucity of evidence in the emergency setting due to the context-sensitive nature of the procedure. However, the foundation for improved outcomes is the same as for elective surgery, with the key elements being the attenuation of the stress response and subsequent organ dysfunction [5]. Opioid analgesics are avoided when possible as it is associated with perioperative cognitive dysfunction and adverse respiratory and gastrointestinal effects [1]. Neuraxial anaesthesia mitigates these risks and has been shown to reduce perioperative complications and fatal outcomes in patients undergoing emergency laparotomies [1]. EA use in emergency laparotomy also reduces abdominal inflammation and facilitates respiratory recovery [5]. Caution is advised when using central neuraxial techniques in patients at risk of haemodynamic collapse or with significant organ dysfunction. Our patient presented with stable haemodynamic and metabolic profiles, prompting the use of TEA for emergency surgery.

While EA use may be regarded as controversial in COPD patients, a study by van Lier et al. showed that the theoretical effect of EA on respiratory muscle function by blunting intercostal nerve conduction was not clinically relevant [6]. EA as compared to spinal anaesthesia causes much fewer changes in inspiratory capacity and expiratory reserve volume [7]. EA also does not affect airway resistance and respiratory gas tensions and it has been shown to improve left ventricular function in high-risk patients by preserving ventricular pulmonary coupling resulting in an improved myocardial oxygen balance [7-9]. GA with tracheal intubation and altered respiratory mechanics may lead to a vulnerable state for right ventricular dynamics. This may be further compounded by the direct cardiodepressant effects of anaesthetic agents on myocardial function [9]. Furthermore, GA affects functional residual capacity, worsens ventilation-perfusion (V:Q) mismatch, and impairs diaphragmatic function [7]. Hence, in high-risk patients such as those with COPD who are at heightened risk of right ventricular dysfunction and adverse ventilatory effects under GA, it is reasonable to advocate for locoregional techniques to be implemented.

The beneficial effects described above provide a hypothesis for expecting improved outcomes with EA use in emergency abdominal surgery. A Danish cohort study concluded that EA uses for emergency laparotomy resulted in decreased morbidity and mortality [10]. Basar et al. performed a continuous spinal anaesthetic (CSA) as the sole technique in 21 elderly high-risk patients (mean Portsmouth Physiological and Operative Severity Score for the Enumeration of Mortality and Morbidity (P-POSSUM) score of 20.5%) undergoing abdominal surgery (ileostomy, colectomy, small bowel resection) and concluded that none of the patients who successfully underwent CSA required level 3 (ICU) care postoperatively. The average length of stay in critical care for this cohort receiving GA for emergency laparotomy in the UK is three days.

Similarly, a study conducted in Italy at the start of the coronavirus disease 2019 (COVID-19) era revealed the feasibility of performing awake major abdominal surgery under EA [2]. Thirteen patients assessed as frail with multiple comorbidities presented for undeferrable surgery at a time when ICU-bed availability was scarce. It was presumed that GA would have exacerbated their clinical condition and would mandate postoperative ICU admission. All patients in this cohort successfully underwent open laparotomy with EA and light sedation as the sole anaesthetic technique, thereby mitigating the need for postoperative ICU care. No complications of EA were encountered, and no adverse events occurred during the perioperative period as a whole. This ICU-preserving approach proved to be feasible and safe for emergency laparotomy at a time of severe ICU bed shortages.

There are case reports of the successful use of non-invasive ventilation (NIV) intraoperatively in patients with respiratory disease, many of whom were dependent on oxygen therapy or had used NIV devices at home for respiratory support. Two such case reports describe the use of NIV in a patient with COPD and chronic type II respiratory failure [11] and the use of continuous positive airway pressure (CPAP) ventilation in a patient with type I respiratory failure [12] both undergoing lower limb surgery. In these cases, the use of NIV mitigated the need for mechanical ventilation in high-risk patients.

A recent systematic review highlights the potential role of intraoperative NIV in patients with respiratory compromise undergoing surgery using neuraxial anaesthesia [7]. These benefits include the reversal of alveolar hypoventilation, prevention or treatment of respiratory failure in “at risk” patients, and improvement of V:Q mismatch [7]. However, caution is warranted when using NIV in certain instances: patients with altered levels of consciousness, severe respiratory compromise warranting immediate intubation, gastric distention, non-cooperative patients, and inability to clear respiratory secretions [7]. In some of these instances, the use of a high-flow nasal cannula device may provide some level of support while obviating some potential problems. At present, in the absence of well-designed randomized controlled trials, judicious use of NIV in combination with neuraxial anaesthesia is recommended [7]. Additionally, the effect of NIV on gastric distention in a patient undergoing laparotomy would preclude its use. As our patient was undergoing an explorative laparotomy for bowel obstruction supplemental, NIV was not considered.

Considering our patient’s comorbidities and current functional status, his risk for significant morbidity and mortality was high. His estimated mortality using the NELA risk adjustment model was 22.1%; a mortality of more than 10% mandates ICU admission [1]. Using the P-POSSUM risk prediction model, he had an estimated 96.8% morbidity risk (haemorrhage, infection, wound dehiscence, anastomotic leak, thrombosis, cardiac failure, impaired renal function, and hypotension). His Assess Respiratory Risk in Surgical Patients in Catalonia (ARISCAT) score for postoperative pulmonary complications was 42% (respiratory failure, infection, pleural effusion, atelectasis, pneumothorax, bronchospasm, and aspiration pneumonitis).

## Conclusions

In a resource-constrained environment, ICU bed shortages are common. However, emergency surgery must proceed, and the anaesthesia technique must be tailored to enable safe surgery while mitigating perioperative risk. EA proved to be a feasible and safe option for emergency abdominal surgery. This technique can be considered in select patients with relation to ICU-bed scarcity and complex comorbidities, with specific reference to chronic respiratory conditions. The supplemental use of NIV with EA should be considered judiciously based on the surgery performed and the patient’s clinical condition.
